# Balancing Routine and Pandemic: The Synergy of India’s Universal Immunization Program and COVID-19 Vaccination Program

**DOI:** 10.3390/vaccines11121776

**Published:** 2023-11-28

**Authors:** Pawan Kumar, Ashish Birendra Chakraborty, Suhas Dhandore, Pritu Dhalaria, Ajeet Kumar Singh, Disha Agarwal, Kapil Singh, Pretty Priyadarshini, Paras Jain, Vidushi Bahl, Gunjan Taneja

**Affiliations:** 1 Immunization Division, Ministry of Health & Family Welfare, New Delhi 110011, India; 2 Immunization Technical Support Unit, Ministry of Health & Family Welfare, New Delhi 110070, India; 3 Bill & Melinda Gates Foundation, New Delhi 110067, India

**Keywords:** pandemic, COVID-19, Universal Immunization Program (UIP), vaccination, zero

## Abstract

The COVID-19 pandemic posed substantial challenges to healthcare systems globally and severely disrupted essential health services, including routine immunization programs. In India, these disruptions were exacerbated due to the sudden emergence of the pandemic and lockdown measures, leading to mass migrations and a shortage of healthcare workers. Caregivers’ concerns about routine immunization sessions further compounded the problem, resulting in a sharp increase in zero-dose children. This review paper examines India’s strategies for conducting one of the world’s largest COVID-19 vaccination programs while effectively restoring and perpetuating its Universal Immunization Program (UIP). The UIP played a pivotal role in sustaining immunization services during the pandemic, ultimately improving immunization coverage compared to pre-pandemic levels. India’s accomplishments in this regard are highlighted through key performance indicators, the reach of immunization services, a reduction in zero-dose children, and antigen-wise coverage. The paper also discusses the successful integration of COVID-19 vaccination within the UIP framework, underscoring the significance of existing infrastructure, technology, and capacity building. India’s dedication to concurrently managing routine immunization and COVID-19 vaccination showcases the adaptability and resilience of its healthcare system. India’s journey serves as a global example of efficient mass immunization during challenging times, emphasizing the importance of political will, healthcare infrastructure investment, skilled healthcare workforces, and comprehensive vaccination programs. In a world grappling with the dual challenge of COVID-19 and routine immunization, India’s experience provides a roadmap for strengthening healthcare systems and promoting public health as the critical agenda in challenging times.

## 1. Introduction

The COVID-19 pandemic led to significant challenges globally and the impact on providing essential health services was profound [1,2,3,4]. In 2021, the pandemic at its peak disrupted essential services (SDG 3.8.1) in 92% of countries, and in 2022 the disruptions were still reported in 82% of countries [5,6,7,8]. The pandemic’s emergence and subsequent lockdowns, particularly in urban areas, led to mass migrations, with individuals in informal settlements and slums returning to rural hometowns [9,10,11]. This was compounded by a shortage of healthcare workers as they were diverted to patient care services along with low vaccine uptake as parents avoided routine immunization (RI) sessions due to fear of their children getting infected. Consequently, routine immunization programs encountered extensive disruptions which led to a steep rise in the number of zero-dose and dropout children as per the WHO/UNICEF Estimates of National Immunization Coverage (WUENIC) 2020 and 2021 Report [12]. While global efforts to restore immunization coverage to pre-pandemic levels are still underway in most countries, India continues to set a precedent through its strong Universal Immunization Program (UIP).

The Ministry of Health and Family Welfare (MoHFW) in India responded to the pandemic with a robust pandemic control and mitigation plan of unprecedented scale. Along with other pandemic control measures, India initiated the COVID-19 vaccination drive within a year of the pandemic and successfully carried out one of the world’s largest COVID-19 vaccination drives [13]. This was a testament to India’s commitment to universal health coverage alongside the resumption of a routine immunization program. This paper aims to explore India’s strategies in rolling out the world’s largest COVID-19 vaccination drive and understand how the UIP and its implementation structure were able to sustain the delivery of routine immunization services to the last mile. The UIP supported the sustenance of immunization services during the pandemic and enabled surpassing of the pre-pandemic levels for most of the vaccines [12,14].

### Performance of India’s RI Program during the COVID-19 Pandemic

The performance of India’s routine immunization program was assessed based on the 2022 WUENIC Report. Key performance indicators like the overall reach of immunization services, the burden of zero-dose children, coverage of measles- and rubella-containing vaccine, and breadth of protection (BOP) have been utilized to assess the overall performance of the country’s immunization program [14]. Below are some of the key focus areas and findings that emerged out of the review of WUENIC estimates. This paper also tries to capture how the robust structure of the UIP was leveraged to successfully carry out one of the world’s largest COVID-19 vaccination drives.

## 2. Reach of Immunization Services

Vaccination against diphtheria, pertussis, and tetanus (DPT) is used as the global marker for immunization coverage or the reach of immunization services [15,16,17,18,19,20]. In India, the reach of immunization services increased to 20.9 million children in 2022 as compared to 19.2 million children in 2021 i.e., 1.7 million more children were reached with the third dose of DPT-containing vaccine in 2022 as compared to 2021. During the same reference period, global immunization services reached 4 million more children in 2022 compared to 2021 i.e., in 2022, around 110 million children were reached with the 3rd dose of DPT-containing vaccine, as compared to 106 million children in 2021. In total, India accounted for a 40% increase in global immunization services in the same period [12,14]. It is noteworthy that India contributes to 17.6% of the world’s total cohort of surviving infants [21].

In India, in 2022, around 1.6 million children were under-vaccinated (i.e., did not receive a third dose of DPT-containing vaccine), which is substantially lower than the number of under-vaccinated children in 2021 (3.4 million) and pre-pandemic levels of 2019 (2 million). On comparing with global performance, it is seen that in 2022, 20.5 million children were under-vaccinated, compared to 24.5 million under-vaccinated children in 2021 and 18.4 million under-vaccinated children in 2019 (pre-pandemic levels). On comparing India’s performance with the global performance, it is evident that although the world is still struggling to reach the pre-pandemic levels of immunization coverage, India has not only reached the pre-pandemic levels but has rather improved [14].

### 2.1. Zero-Dose (ZD) Children

**Figure 1:**
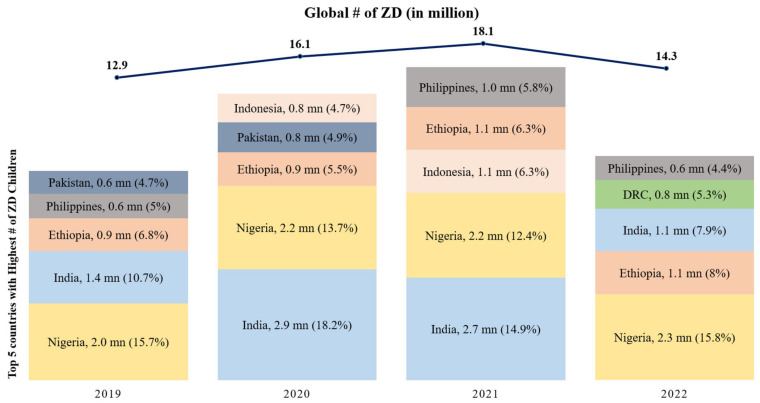
Top 5 countries with the highest number of Zero-Dose children since 2019 (in millions) (Source: WUENIC 2019–2022).

Figure 1 shows the percentage of zero-dose children in different countries. In 2021, India found itself among the countries with the highest number of zero-dose children due to disruptions in routine immunization services triggered by the COVID-19 pandemic. However, the year 2022 marked a significant turning point, reflecting a remarkable reduction in this number. According to the latest WUENIC report in 2022, the count of zero-dose children in India, meaning those who had not received even a single dose of DPT-containing vaccine within their first year of life, dropped substantially from 2.7 million in 2021 to 1.1 million in 2022, even lower than the pre-pandemic levels of 2019 when the count stood at 1.4 million.

To contextualize this progress on a global scale, Figure 1 highlights the top five countries with the highest number of zero-dose children since 2019. In 2019, India had 1.38 million zero-dose children, ranking as the second-highest contributor globally at 10.7%, after Nigeria. During the COVID-19 pandemic, India faced the highest number of zero-dose children in 2020 and 2021, contributing 18.2% and 14.9%, respectively. However, in 2022, India’s efforts led to a significant reduction in zero-dose children, and it moved to the third spot, contributing 7.9% to the global burden. Nigeria, Ethiopia, DRC, and the Philippines were among the other countries in the top five. These data underscore India’s substantial progress in immunization and its role in the global context, alongside these other nations [12].

### 2.2. Antigen-Wise Coverage

**Figure 2:**
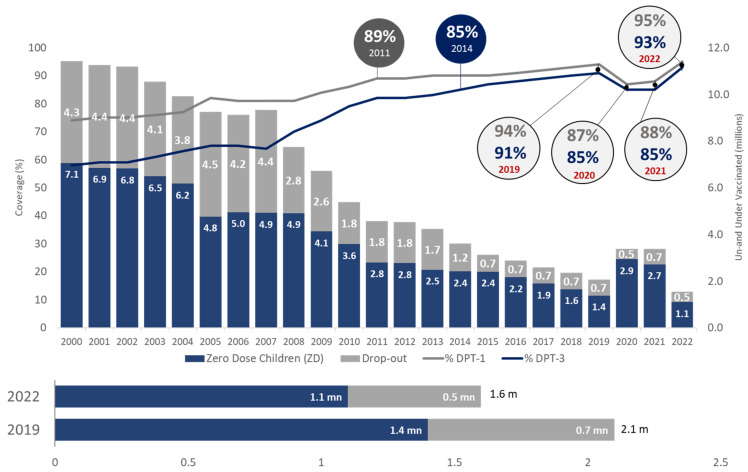
Trend of immunization coverage in India (Source: WUENIC).

According to the WUENIC estimates, 2022, for India, the year-wise coverage trend for the key antigens is tabulated below:

The Table 1 latest estimates depict that except for BCG, India has achieved the pre-pandemic coverage levels of measles-containing vaccine (MCV-1) and rubella-containing vaccine (RCV-1) and has surpassed the pre-pandemic (2019) coverage levels for most of the other vaccines i.e., DPT-1, DPT-3, oral polio vaccine (OPV-3), inactivated polio vaccine (IPV-1), MCV-2, hepatitis B (Hep-B) birth dose, rotavirus vaccine (RVV-3), and pneumococcal conjugate vaccine (PCV-3). Figure 2 shows in 2022, DPT-3 coverage soared to an impressive 93%, surpassing the pre-pandemic peak of 91% recorded in 2019. Overall, the number of zero-dose (1.1 mn) and dropout (0.5 mn) children reduced to 1.6 million in 2022, much lower than 2.1 million in 2019.

**Figure 3:**
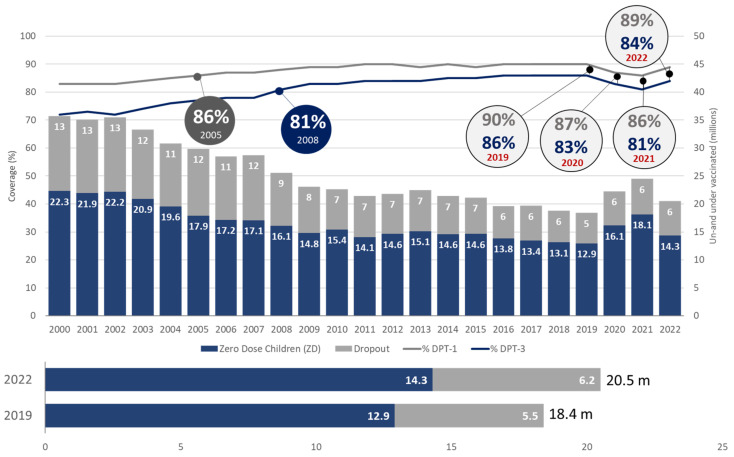
Global trend of immunization coverage (Source: WUENIC 2019–22).

Table 2 and Figure 3 show that, globally, pre-pandemic coverage levels were achieved for IPV-1, MCV-2, Hep-B birth dose, RVV last dose, and PCV-3 only. The global DPT-1 and DPT-3 coverage increased in 2022 as compared to 2021, however, it failed to achieve the pre-pandemic levels. In 2022, DPT-3 coverage soared to an impressive 93%, surpassing the pre-pandemic peak of 91% recorded in 2019. Overall, at a global level the number of zero-dose (14.3 mn) and dropout (6.2 mn) children increased to 20.5 million in 2022, higher than 18.4 million in 2019.The trend is shown in the table and graph below:

### 2.3. Measles and Rubella (MR) Vaccine Coverage

**Figure 4:**
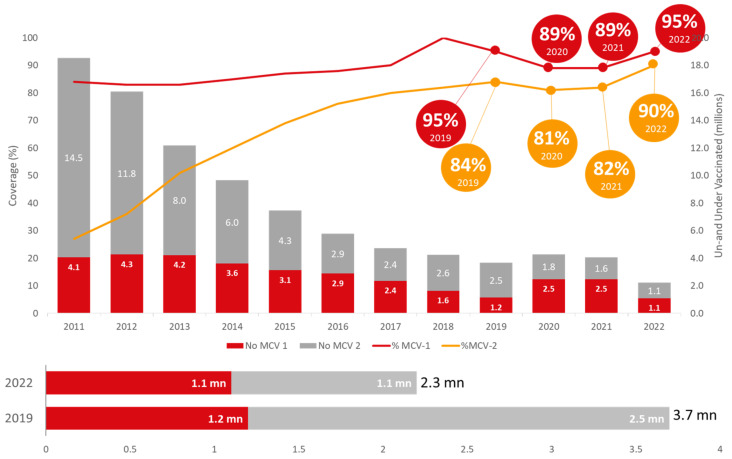
Trend of measles-containing vaccine in India (Source: WUENIC 2019–2022).

It is of concern that, globally, vaccination against measles has not recovered to pre-pandemic levels as well as other vaccines, putting an additional 32.6 million children at risk of measles infection. Low-income countries have not recovered well, however, there has been a considerable recovery in middle-income countries like India. Figure 4 shows in view of India’s ambitious target of eliminating measles and rubella by 2023, it is noteworthy that the coverages of measles- and rubella-containing vaccines’ 1st and 2nd doses have been reported to be 95% and 90%, respectively, in 2022. The number of children not receiving MCV-1 (1.1 mn) and not receiving MCV-2 (1.1 mn) in total was 2.3 million, which is substantially lower than even the pre-pandemic levels (2019).

**Figure 5:**
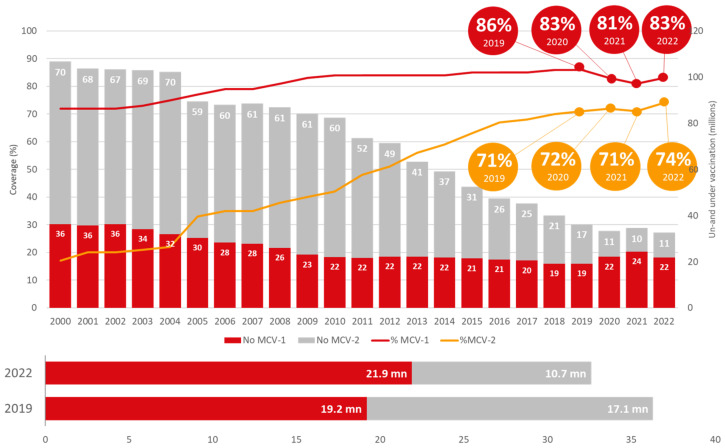
Global trend of measles-containing vaccine (Source: WUENIC 2019–2022).

The Figure 5 shows globally, the coverage of the 1st dose of MCV increased to 83% in 2022 from 81% in 2021 but remained lower than the 86% achieved in 2019. The coverage of the 2nd dose of MCV, although sub-optimal, increased from 71% in 2019 to 74% in 2022. In total, 32.6mn children were under- or unvaccinated for measles in 2022, which is lower compared to the 2019 pre-pandemic level (36.3 mn).

### 2.4. Breadth of Protection

**Figure 6:**
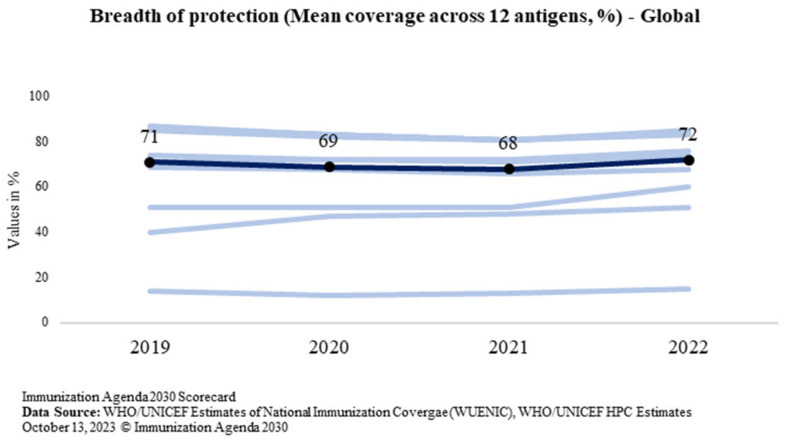
Global trend of the breadth of protection (Source: WUENIC).

Breadth of protection is a combination of the number of vaccines in a country’s immunization program and the coverage achieved for each vaccine [5]. Globally, it is measured for all countries as the mean coverage for all WHO-recommended vaccine antigens which is calculated by including the 13 antigens, regardless of whether the antigen has been introduced in the country—diphtheria, tetanus, pertussis, polio, measles, hepatitis B (Hep-B), rubella, Haemophilus influenzae type B (Hib), measles 2nd dose, rota, pneumococcal conjugate vaccine (PCV), inactivated polio vaccine (IPV), and human papilloma virus (HPV) [22]. The global trend of the breadth of protection is shown in Figure 6.

**Figure 7:**
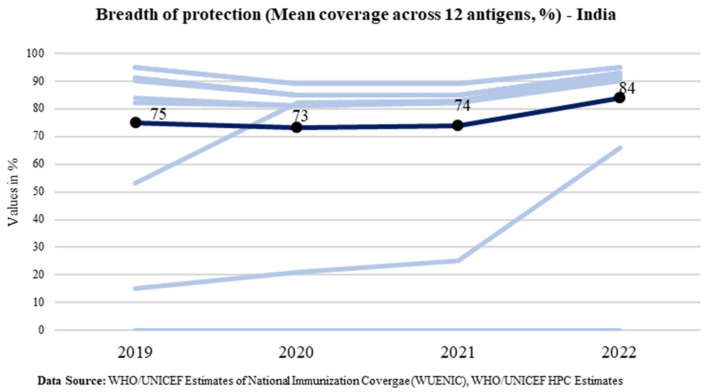
Trend of the breadth of protection in India (Source: WUENIC).

While the BOP at the global level has marginally increased from the pre-pandemic level of 71% in 2019 to 72% in 2022, India has bounced back in 2022 to increase the protection for all antigens under the program for every child of the country. The BOP reported in 2022 is 84% which is 9 percentage points above the pre-pandemic level in 2019 (75%). India’s trend of the breadth of protection is shown in Figure 7. It shows that the breadth of protection increased to 84% from 75%.

**Figure 8:**
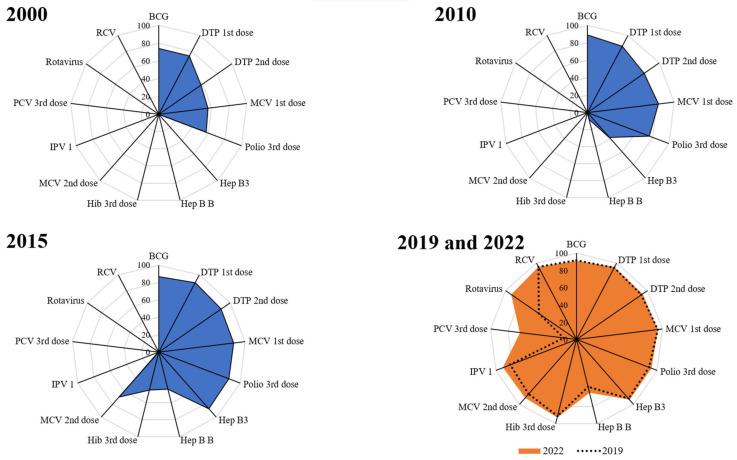
Spidergrams depicting the breadth of protection from vaccines.

The spidergram (Figure 8) depicts the expanding breadth of protection over the years, specifically in comparison to the pre-pandemic levels. Since 2014, India has expanded the basket of vaccines provided under the UIP by introducing six new vaccines—inactivated polio vaccine (IPV), measles-containing vaccine (MCV), rotavirus vaccine (RVV), pneumococcal conjugate vaccine (PCV), adult Japanese encephalitis (JE) vaccine, and tetanus and adult diphtheria vaccine (Td). It is evident from the analysis depicted below that the introduction of newer vaccines under the UIP over the years has led to an increase in the breadth of protection. This expanded breadth of protection is a critical incremental improvement in expanding vaccination services to everyone [12]. Any new vaccine introduction contributes to the overall system strengthening, ensuring the delivery of services. It provides an opportunity for training, capacity building, community sensitization, cold chain assessment, and programmatic improvements and scale-up [23,24,25,26]. In recent years, India has introduced six new vaccines under the program, which made the system more robust and resilient and also supported the quick and successful rollout of the COVID-19 Vaccination Program as well as recovering routine immunization services immediately after the pandemic.

It is particularly noteworthy that the pneumococcal conjugate vaccine was expanded to cover the entire nation during the COVID-19 pandemic. India began a phasic introduction of the pneumococcal conjugate vaccine (PCV) in 2017 in five states—Bihar, Himachal Pradesh, Madhya Pradesh, Rajasthan, and Uttar Pradesh. But the real breakthrough happened from 2021–2022 when PCV was expanded nationwide as part of the UIP. This was a courageous step taken by the Government of India (GoI) when the pandemic was at its peak [27].

## 3. Factors Driving Sustenance of UIP Performance during the Pandemic

The mammoth task of immunizing 132 million children, including 26 million infants, and additionally 30 million pregnant women every year, involves the synchronized efforts of countless healthcare workers, an efficient supply chain, electronic records, and meticulous logistical planning. The suspension of immunization sessions during the early phase of the pandemic, i.e., March 2020, led to the disruption of immunization services [28,29,30,31,32,33]. However, recognizing the distressing consequences of disruption, the GoI reiterated immunization as an essential health service in April 2020, providing states with guidelines to recommence routine immunization services in alignment with the COVID-19 control and mitigation plan. As of June 2020, India began a phased reopening of its economy, with the resumption of immunization activities in accordance with local COVID-19 infection rates and restrictions [9].

### Special Vaccination Drive—Intensified Mission Indradhanush and Outreach Sessions

Recognizing the urgency to reach and immunize the children who had been left behind, India introduced the Intensified Mission Indradhanush (IMI) 3.0 initiative in early 2021. This attempt aimed to bridge the immunization gaps, encompassing the backlog of missed vaccinations, dropouts, and unvaccinated individuals by focusing on areas where routine immunization services were most affected by the pandemic. Subsequently, another round—IMI 4.0—was conducted in 2022 to catch up on any existing gaps in immunization coverage due to the pandemic. Approximately 6 million children and 1.5 million pregnant women were vaccinated during IMI 4.0. The government’s approach was to focus on high-risk regions and intensify the drive amongst vulnerable populations, including migrants. In 2023, India continued its commitment to immunization and launched IMI 5.0. With its goals to eliminate the highly contagious diseases of measles and rubella, IMI 5.0 is more than just a vaccination drive; it is a visionary pursuit of a measles-free India. The conduction of such intensified immunization activities showcased a nation that not only confronted challenges head-on but transformed them into opportunities for growth [34].

## 4. India’s COVID-19 Vaccination Program Achievements

The urgency with which the GoI responded to the pandemic was the cornerstone for combating the posed threat and building trust in its citizens. As the COVID-19 vaccines were being developed by several manufacturers across the globe, including India, the Indian government simultaneously started strategizing for the rollout of the National COVID-19 Vaccination Program. The government established a National Expert Group on Vaccine Administration for COVID-19 (NEGVAC) within 6 months of the declaration of the COVID-19 pandemic as a Public Health Emergency of International Concern (PHEIC), to meticulously guide all facets of the country’s COVID-19 vaccination initiative [35,36].

The National COVID-19 Vaccination Program was launched by the Hon’ble Prime Minister of India on 16 January 2021. The vaccination strategy was in alignment with the global scientific evidence and was paced considering the availability of resources. Initially, only limited vaccine doses were available and hence healthcare workers (HCWs) were the first to be vaccinated to manage the pandemic at its forefront. Subsequently, as the drive progressed, the vaccine was made available to the next most susceptible cohort—the elderly population. It was further expanded in a phased manner to include the population aged 18 years and above and eventually to the population 12 years and above [35,37]. A precautionary dose was made available for the high-risk cohort, as evidence of the vaccine’s effectiveness emerged and the resources were secured. It is noteworthy that all COVID-19 vaccine doses were provided free of cost to the entire target population. Private hospitals’ participation was also ensured to increase the pace of vaccination.

India witnessed vaccination of the highest number of beneficiaries covered anywhere in the world. During its journey of more than 2 years, unparalleled milestones were achieved by the administration of 10.3 million, 13 million, and more than 2.5 crore beneficiaries on singular days. India achieved a major landmark of administering 100 crore vaccines in a short span of 9 months since the drive began and 200 crore COVID-19 vaccines on 17 July 2022. As of 31 August 2023, more than 220.67 crore vaccine doses have been administered across the country among the eligible populations aged 12 years and above [35].

Har Ghar Dastak (*Jan Bhagidari Andolan*)—a month-long door-to-door campaign—was launched in November 2021 and one of the innovations that ensured people’s participation in the COVID-19 Vaccination Program. The objective was to vaccinate all eligible beneficiaries with the first dose of vaccine including those who were either left out or dropped out through rigorous household visits by frontline health workers. This led to a massive increase in vaccination coverage and mobilizing communities and hence another round of Har Ghar Dastak 2.0 was launched on 1 June 2022 to accelerate the pace and intensify the COVID-19 vaccination until 31 July 2022 [38].

The vaccination program has been constantly informed by scientific evidence and the ground situation of the pandemic, providing the best possible recommendations and guidance for implementation. Over time, strategic interventions were instituted to make the vaccination program and its process more people-centric, convenient, and pragmatic. While the central government assumed a pivotal role in strategic planning, procurement, distribution, and price determination, state governments took charge of executing the campaign. The central government’s ownership in vaccine procurement yielded economies of scale, enabling cost-effective bulk orders [36].

## 5. Leveraging UIP’s Structure for Rolling out National COVID-19 Vaccination Program

One of the key strategies that ensured the COVID-19 Vaccination Program was successful remains resorting to the existing UIP framework. Over the years, many health-system-strengthening initiatives, the introduction of new vaccines, regular supplementary immunization activities, and other catch-up vaccination campaigns have strengthened the UIP and empowered it with the adaptability to handle large-scale vaccination programs. Building upon the UIP’s strengths, some key areas such as human resources and frontline health workers, capacity building and training methodology, vaccines and logistic supply and distribution mechanisms, information, education, and communication (IEC), monitoring, supervision, and review platforms were leveraged to roll out the National COVID-19 Vaccination Program. Some of the major initiatives that paved the way for the successful implementation of the drive are enumerated below [39,40].

### 5.1. Human Resource and Capacity Building

The national, state, and district immunization officers and medical officers in charge (MOICs) at the block level steered the implementation of the COVID-19 Vaccination Program in the country. The vaccinators and auxiliary nurse midwives (ANMs) under the UIP were engaged in COVID-19 vaccination and the community mobilization was carried out by accredited social health activists (ASHAs). The rollout of COVID-19 vaccination required frequent capacity building of all the staff engaged. The existing cascading model of training with the support of immunization partners was leveraged to carry out frequent capacity building at all levels of implementation [41].

### 5.2. Cold Chain, Vaccine, Logistic Supply, and Distribution System

The country has an extensive cold chain system with more than 29,000 cold chain points. India skillfully harnessed its pre-existing cold chain network to ensure the maintenance of the cold chain for COVID-19 vaccines. This strategic use of infrastructure guaranteed vaccine potency and effectiveness throughout from manufacturing to administration. The already established structure of state vaccine stores, regional vaccine stores, district vaccine stores, and cold chain points along with the Government Medical Store Depot (GMSD) was utilized to store the vaccines and undertake distribution to the field level [40].

### 5.3. Electronic Vaccine Intelligence Network (eVIN)

The remarkable synergy between India’s robust cold chain system and innovative technologies like eVIN has been the linchpin of the nation’s successful COVID-19 Vaccination Program. It has allowed India to efficiently navigate one of the most extensive vaccination programs globally, covering an incredibly diverse and vast population. In essence, India’s ability to seamlessly integrate its pre-existing infrastructure with cutting-edge technologies showcases its unwavering commitment to public health. This innovation revolutionized the reception, storage, and dissemination of vaccines, through the management of vaccine stocks and continuous temperature monitoring in real time at every cold chain point. eVIN ensures that there is uninterrupted tracking of vaccine supply and its replenishment. This well-established system has played a pivotal role in the success of India’s COVID-19 Vaccination Program [42,43].

### 5.4. COVID Vaccine Intelligence Network (Co-WIN)

The successful implementation of eVIN lies at the core of conceiving and scaling up Co-WIN—an innovative digital platform dedicated to registering and managing COVID-19 vaccinations. This platform has facilitated immunization at an unprecedented scale in a remarkably short span. Developing an effective digital health system catering to over a billion beneficiaries might seem daunting, however, India’s response to the challenge was resounding. India developed the Co-WIN platform within a mere three months, amidst the constraints posed by the COVID-19-pandemic-induced lockdown. This impressive feat facilitated the administration of 2 billion COVID-19 vaccine doses in under 24 months, safeguarding the well-being of more than 95% of eligible adults. Co-WIN’s success serves as a beacon for India’s progressive digitization of its healthcare systems, optimizing efficiency and extending healthcare access to remote areas. The roots of the Co-WIN platform can be traced back to the learnings derived from eVIN. This seamless transition from eVIN to Co-WIN highlights India’s capability to build upon existing frameworks to achieve greater technological feats [44,45].

### 5.5. Adverse Events Following Immunization (AEFI) Surveillance and SAFE-VAC

AEFI surveillance structure was expanded with the inclusion of more experts, enhanced capacities, increased frequency of meetings, and expanded roles and responsibilities. Moreover, as part of the process to strengthen the reporting system for adverse events following immunization, Surveillance, and Action for Events following Vaccination (SAFE-VAC)—a digital platform for reporting serious/severe AEFI cases following routine immunization—was developed and introduced in 2019 under the UIP. During the pandemic, SAFE-VAC was integrated with the Co-WIN portal for reporting AEFIs in India. This integration served several important purposes, including streamlining the process of reporting AEFIs, consolidating data from the COVID-19 Vaccination Program, and emphasizing the importance of monitoring and managing potential adverse events alongside successful vaccination implementation [46].

### 5.6. Advocacy and Community Mobilization

The leadership of the country has always been a big advocate for childhood immunization. For COVID-19 vaccination, the Prime Minister of India advocated the importance of COVID-19 vaccines through platforms like ‘Mann ki Baat’—a monthly radio program through which the Prime Minister was able to connect with the Indian citizens, share the country’s vision, and foster participation. Moreover, all the existing channels like social media and local and print media were used to escalate vaccine demand and dispel myths about COVID-19 vaccines [35].

### 5.7. Review Platforms and Monitoring Mechanism

Under the UIP, there is a defined and established program review structure of the State Steering Committee, State Task Force, District Task Force, and Block Task Force across all 36 states and Union Territories (UTs). The composition, standard operating procedures, mechanism, and frequency of meetings are pre-defined for all these review platforms. These pre-existing platforms were expanded with inclusive representation and enhanced roles for reviewing the COVID-19 Vaccination Program. Existing routine immunization monitoring platforms were used to collate monitoring data up to the block level on a real-time basis. These data were reviewed during evening review meetings to guide corrective actions. Monitoring feedback guided corrective actions through capacity building and strengthening the program further [13,41].

### 5.8. Partnerships

The importance of collaboration and the power of the collective for all stakeholders were well established and pursued. Efforts were made for coordination and synergy towards successful planning and implementation of COVID-19 vaccination. The roles and responsibilities of each partner and department were illustrated and tapped to their full potential. Convergence of medical college representatives, professional bodies such as the Indian Medical Association (IMA), Indian Academy of Paediatricians (IAP), representatives at the district level, developmental partners including the WHO, UNICEF, UNDP, BMGF, voluntary organizations—National Cadet Corps, National Service Scheme, Nehru Yuva Kendra, non-government organizations—Lions International, Rotary International, Red Cross, Civil Society Organizations, etc. was operationalized [13,41].

## 6. Discussion

The global impact of the COVID-19 pandemic on routine immunization programs has led to huge disruptions since its onset [11,15,16,17,18,19,20]. Qualitative insights reveal various factors affecting these programs, emphasizing the need for comprehensive strategies to address these disruptions. Data across sources and regions demonstrated the pandemic’s significant impact on vaccination services, highlighting the widespread disruption of essential health services [7,8,9,10]. Outreach services, particularly vital in low- and middle-income countries, encountered substantial challenges due to restricted access and apprehension among the population [33,47]. This disruption places the vulnerable communities primarily relying on these services at heightened risk of vaccine-preventable diseases. The imperative now lies in targeted catch-up efforts to restore full immunization coverage (FIC). Most countries are currently striving to restore immunization coverage to pre-pandemic levels. Prioritizing catch-up vaccination strategies, especially for vulnerable communities and those potentially at risk, is vital. The Immunization Agenda 2030 provides a roadmap for sustaining vaccination gains once the pandemic recedes, preparing countries for future catastrophes and building sustainable systems [18,47]. The causes of delayed or interrupted immunizations were diverse, including parental fears, restricted movements, healthcare personnel prioritizing COVID-19 response, and logistical challenges. The COVID-19 pandemic has reverberated globally, highlighting the vulnerabilities within public health systems [11]. This disruption underscores the importance of fortified healthcare infrastructures capable of withstanding challenges and safeguarding populations. It also highlights the interconnectedness of health services and the fragility of comprehensive healthcare [19]. The crisis catalyzed innovative strategies, emphasizing the significance of equitable access to healthcare. It offers an opportunity to reshape healthcare systems, bolstering resilience and adaptability. Preserving high immunization coverage is crucial for preventing post-pandemic outbreaks of vaccine-preventable diseases [7,25]. The global effort to simultaneously sustain COVID-19 vaccination and routine immunization has been challenging, however, countries are striving to balance both and ensure recovery [7].

India’s response to the pandemic serves as an outstanding example of strategic resilience. The government efficiently vaccinated the world’s second-largest population at an unprecedented pace, outpacing many other nations. This remarkable achievement was made possible by India’s enduring commitment to building a robust UIP system over the years, alongside fortifying its broader healthcare infrastructure [40]. The phased resumption of immunization, adhering to WHO guidelines and synchronized with economic reopening, is commendable. India’s commitment to healthcare was further exemplified by the simultaneous launch of the COVID-19 Vaccination and Routine Immunization Programs, showcasing a comprehensive approach to public health. The UIP’s framework optimized existing infrastructure, skilled personnel, and established protocols, facilitating the efficient distribution, storage, and administration of routine and COVID-19 vaccines. This process of adaptation and optimization was a window of opportunity to identify the gaps in the health infrastructure and cold chain systems. The government seized this opportunity to dissolve the systemic gaps. During the pandemic, the government invested in infrastructure, cold chain systems, and logistics that played a vital role in maintaining the integrity of COVID-19 vaccines. These improved systems are now being availed for the routine immunization program, further adding to its robust fabric.

India’s digital health journey is itself an exemplar for the world and the government brought digital innovations to its forefront for driving COVID-19 vaccination and pandemic management through Co-WIN. eVIN was instrumental in conceptualizing and scaling up Co-WIN and India continues to reinforce its digital footprint. India piloted yet another digital platform in 2023—U-WIN—to register and track all beneficiaries and ensure no one was left or dropped out of the system. The lessons from Co-WIN led to the development of U-WIN—a name-based immunization registry system that will serve as a single source of information across all states and UTs. It will cater to all the needs of the UIP through interlinkages and interoperability with other immunization data sources and portals [48]. The three digital innovations—eVIN (2015), Co-WIN (2021), and U-WIN (2023)—have revolutionized the immunization program in India and exemplify India’s commitment to immunize one of the largest cohorts in the world [49].

India’s adeptness in executing large-scale vaccination programs, the proficiency of its healthcare workforce, and the comprehensive UIP framework were pivotal in ensuring the safe and effective delivery of COVID-19 vaccines [35]. The sustained efforts to foster public awareness and trust in vaccines significantly elevated the rate of vaccination. The commitment of the Indian government was evident in strategic resource allocation, collaboration, and vaccine supply, propelling the triumphant trajectory of the COVID-19 Vaccination Program [25]. India serves as a global exemplar of proficient mass immunization, and the journey through these challenges reveals India’s remarkable capacity for adaptation, resilience, and innovation [49]. India’s efforts have been sustained through transparent, coherent, and timely communication from public health and immunization providers, adhering to the WHO’s guidance that emphasizes the continuation of essential services alongside COVID-19 mitigation efforts to uphold public trust and minimize health impact. As societies worldwide strive to regain normalcy, India’s experience serves as a guiding light for safeguarding healthcare systems and an indelible mark on the journey toward a healthier world.

The COVID-19 cases and deaths have drastically reduced around the world; however, millions of people continue to be infected with SARS-CoV-2 and the number of deaths still accounts for thousands a week [50]. In India, the numbers of COVID-19 cases and vaccinations have reduced drastically to a few hundred and states have been empowered to make decisions regarding vaccine procurement, delivery, and implementation based on their needs. To date, ~90% of eligible beneficiaries have received both doses of COVID-19 vaccine in India [51]. Meanwhile, this year the government also reviewed its public health preparedness for the management of COVID-19 and the government continues to emphasize the ‘Five-fold Strategy of Test–Track–Treat–Vaccinate, and Adherence to COVID-Appropriate Behaviour’ irrespective of the new COVID variants. States have also conducted a series of mock drills in health facilities to assess the readiness of infrastructure and ramp up testing and vaccination [52].

India confronts substantial challenges in upholding routine immunization coverage, particularly as the world’s largest birth cohort. Even minor disruptions can trigger far-reaching consequences. The COVID-19 pandemic has exacerbated these challenges, introducing disparities in routine immunization coverage. Stringent lockdown measures, implemented to curb the virus’s spread, coupled with the mass urban-to-rural migration have left a considerable number of under- and unimmunized children [9]

The increased workload and the prioritization of COVID-19 vaccination over routine immunization have impacted the execution of planned immunization sessions. Despite India’s commendable achievements, the pursuit of the final goal of measles and rubella (MR) elimination has hindrances like vaccine hesitancy, unexpected disruptions, etc. Addressing these challenges necessitates a comprehensive and targeted approach to fortify India’s immunization programs needs.

This year, the WHO has also updated its Strategic Preparedness and Response Plan (SPRR) for 2023–2025 with the objective of supporting countries to transition from the emergency preparedness phase to long-term disease management. It emphasizes five core competencies of effective health emergency preparedness, response, and resilience (HEPR) that have been mapped against ten COVID-19 operational pillars [50]. Countries can adapt these strategies according to their needs, priorities, and futuristic projections and eventually gauge the potential to integrate them into life course vaccinations.

## 7. Conclusions

The COVID-19 pandemic has highlighted the importance of routine immunization and the resilience of healthcare systems. India’s experience serves as a beacon of hope and guidance for the world, emphasizing the need for strong political will and commitment, stewardship, investments in healthcare infrastructure, skilled healthcare workforces, and comprehensive vaccination programs. As we continue to navigate the challenges of the pandemic, India’s journey reminds us that public health and immunization are vital components of societal well-being and calls for uninterrupted commitment and innovation.

## Figures and Tables

**Table 1:** Trend of antigen-wise coverage in India (Source: WUENIC 2019–22).

Vaccine	2019	2020	2021	2022
BCG	92	85	84	91
DPT-1	94	87	88	**95**
DPT-3	91	85	85	**93**
OPV-3	90	85	85	**93**
IPV-1	82	81	82	**91**
MCV-1	95	89	89	95
MCV-2	84	81	82	**90**
RCV-1	95	89	89	95
Hep-B birth dose	56	54	55	**63**
RVV-3	53	82	83	**92**
PCV-3	15	21	25	66

**Table 2:** Global trend of antigen-wise coverage (Source: WUENIC 2019–22).

Vaccine	2019	2020	2021	2022
BCG	89	85	84	87
DPT-1	90	88	86	89
DPT-3	86	83	81	84
OPV-3	87	83	81	84
IPV-1	83	80	80	84
MCV-1	86	83	81	83
MCV-2	71	72	71	74
RCV-1	69	68	66	68
Hep-B birth dose (within 24 h of birth)	44	43	42	45
RVV-3 last dose	40	47	48	51
PCV-3	51	51	51	60

## Data Availability

The data are available in the public domain and can be downloaded from: accessed on 10 October 2023 https://immunizationdata.who.int/listing.html?topic=&location=.
